# Prognosis and Survival Modelling in Cirrhosis Using Parenclitic Networks

**DOI:** 10.3389/fnetp.2022.833119

**Published:** 2022-02-21

**Authors:** Han Zhang, Tope Oyelade, Kevin P. Moore, Sara Montagnese, Ali R. Mani

**Affiliations:** ^1^ Network Physiology Laboratory, Division of Medicine, University College London, London, United Kingdom; ^2^Institute for Liver and Digestive Health, Division of Medicine, University College London, London, United Kingdom; ^3^ Department of Medicine, University of Padova, Padova, Italy

**Keywords:** cirrhosis, MELD, network physiology, parenclitic, prognosis, survival

## Abstract

**Background:** Liver cirrhosis involves multiple organ systems and has a high mortality. A network approach to complex diseases often reveals the collective system behaviours and intrinsic interactions between organ systems. However, mapping the functional connectivity for each individual patient has been challenging due to the lack of suitable analytical methods for assessment of physiological networks. In the present study we applied a parenclitic approach to assess the physiological network of each individual patient from routine clinical/laboratory data available. We aimed to assess the value of the parenclitic networks to predict survival in patients with cirrhosis.

**Methods:** Parenclitic approach creates a network from the perspective of an individual subject in a population. In this study such an approach was used to measure the deviation of each individual patient from the existing network of physiological interactions in a reference population of patients with cirrhosis. 106 patients with cirrhosis were retrospectively enrolled and followed up for 12 months. Network construction and analysis were performed using data from seven clinical/laboratory variables (serum albumin, bilirubin, creatinine, ammonia, sodium, prothrombin time and hepatic encephalopathy) for calculation of parenclitic deviations. Cox regression was used for survival analysis.

**Result:** Initial network analysis indicated that correlation between five clinical/laboratory variables can distinguish between survivors and non-survivors in this cohort. Parenclitic deviations along albumin-bilirubin (Hazard ratio = 1.063, *p* < 0.05) and albumin-prothrombin time (Hazard ratio = 1.138, *p* < 0.05) predicted 12-month survival independent of model for end-stage liver disease (MELD). Combination of MELD with the parenclitic measures could predict survival better than MELD alone.

**Conclusion:** The parenclitic network approach can predict survival of patients with cirrhosis and provides pathophysiologic insight on network disruption in chronic liver disease.

## Introduction

The liver is the physiological hub for multiple homeostatic, metabolic, synthetic and immune functions. Thus, patients with liver failure exhibit various neural, renal, cardiovascular, endocrine, and metabolic manifestations. Cirrhosis is a complex disease caused by alcohol, chronic viral hepatitis, fatty liver or other causes (Asrani et al., 2019) and involves multiple organ-systems and functions. Thus, interpretation of organ dysfunction without consideration of the whole system is illogical. This is evident in the relative difficulty in the management of complications of cirrhosis whereby targeting a single organ dysfunction may lead to the dysregulation of other tightly balanced pathways (Harrison, 2018). This makes prediction of treatment response and prognosis especially challenging and further complicates prioritization of liver transplantation (Dutkowski et al., 2015; Jadlowiec and Taner, 2016). The introduction of several prognostic scores and models such as Child-Pugh, MELD, UKELD amongst others is in direct response to the complexity of decompensated cirrhosis and while these models have been useful, various limitations continue to surface (Biselli et al., 2010).

The future of disease diagnosis, management and prognosis will likely benefit from a network physiology approach providing a more global or holistic view of the changes in the physiological interactome leading to disrupted states. Network physiology focuses on complex interactions among diverse organ systems in health and disease (Bashan et al., 2012) and provides a viable alternative to the conventional scoring methods and facilitate the evaluation of organ systems interaction in complex disorders such as cirrhosis. Early work by Asada et al., on critically ill patients in the intensive care unit showed disrupted network of organ systems interaction in non-survivors. In Asada et al. (2016) study, the degree of organ systems interaction was assessed by calculating the correlation between biomarkers (e.g., correlation between creatinine and bilirubin as biomarkers for renal and hepatic function respectively). Then a network was mapped using individual biomarkers as its nodes and correlation coefficients as the edges of the network. Their results showed that in a cohort of critically ill patients, survivors consistently exhibited a higher number of edges and clusters compared to non-survivors in their organ connectivity network structures (Asada et al., 2016). In a recent report, we used a similar approach and showed that functional connectivity of organ systems is significantly disrupted in patients with cirrhosis who did not survive during 12-month follow up (Tan et al., 2020). However, the methodology of these studies is based on correlation analysis of a population of patients and cannot be used for mapping the network connectivity at the level of individual patients. Hence, these reports provide insight about the pathophysiology in general but doesn’t allow clinical application to individual subjects (Asada et al., 2016).

**FIGURE 1:**
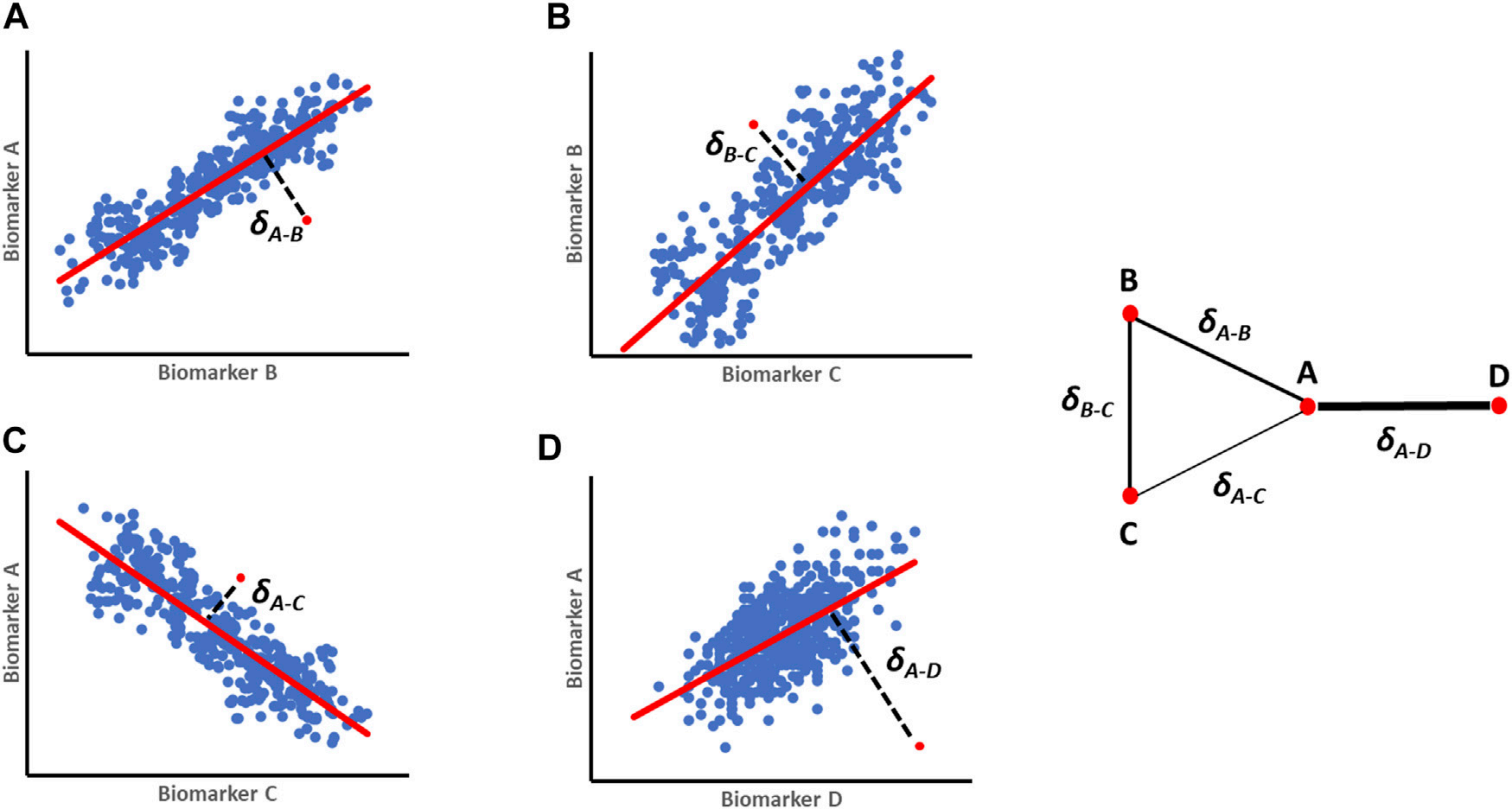
A schematic representation of orthogonal residuals (δ) calculation and translation into parenclitic network. **(A–D)** First regression models are built for pairs of variables (A-B; B-C; A-C and A-D) from a reference population (e.g., survivors, treatment responders etc.). The blue dots represent individual reference data, the red regression lines represent the expected relationship models, while the red dots are individual data of patients being studied. The black lines represent the deviation values (δ). The resulting parenclitic network map of nodes A, B, C and D is presented with edges weighted (in terms of thickness) according to the magnitude of deviations from the models.

The parenclitic network analysis was proposed by Zanin et al. (2014) to create a network from the perspective of an individual subject in a population. Instead of looking at the network of connections in a population, this approach provides a method for mapping a network for each subject, where nodes represent features and links are weighted according to the *deviation* between a subject’s features and their corresponding typical relationship within a studied population (“Parenclitic” mean “deviation” in Greek) (Zanin et al., 2014). In its simplest form, the model can be a simple linear regression between all possible pairs of features in the population, followed by the calculation of deviations between values of a particular subject and pre-constructed reference models (Figure 1). A network map is then constructed for individual subjects whereby each feature represents a node and deviation from the reference model is defined as edges between the nodes. The topological characteristics of the resulting network of individual subject can be used to extract important information about the relationships of the system. Since its first description, parenclitic network analysis has been used both in genetic mapping of cancer (Zanin, 2016; Karsakov et al., 2017; Whitwell et al., 2018), Down syndrome (Krivonosov, 2020), aging (Whitwell et al., 2020) and even criminology (Zanin, 2018) and continues to open up new insights in complex systems.

In the present study we applied a parenclitic approach using standard clinical/laboratory data to map the physiological network of individual patients with cirrhosis. We then tested whether this approach could predict survival independent of current measures of severity of cirrhosis such as MELD and Child-Pugh scores.

## Methods

### Ethics

The study protocol was approved by the Padova Hospital Ethics Committee. All participants provided written informed consent. This study was conducted according to the Declaration of Helsinki (Hong Kong Amendment) and Good Clinical Practice (European) guidelines.

### Patients Cohorts

The study population consists of 106 patients diagnosed with cirrhosis referred to the tertiary referral liver centre of the Clinica Medica V, Padova University Hospital, for formal hepatic encephalopathy assessment. Patients were enrolled between 2009 and 2018 according to the inclusion/exclusion criteria [for more information about patients’ recruitment see Formentin et al. (2021)].

### Inclusion/Exclusion Criteria

Inclusion criteria include confirmed diagnosis of cirrhosis based on clinical manifestations and/or liver imaging. Exclusion criteria includes age under 16 or over 80, hepatocellular carcinoma, severe co-morbidity with short prognosis *per se*, a history of cirrhosis on a transplanted liver, significant head injury, neurological or psychiatric disease other than hepatic encephalopathy, active alcohol misuse or acute infection.

### Follow Up

Patients meeting above inclusion and exclusion criteria were studied retrospectively and further separated into survivor and non-survivor groups by the survival status during the follow-up periods (12 months). Patients who were transplanted due to liver failure were classed as non-survivors as they were in immediate need of a new liver and would not survive without transplantation (Oyelade et al., 2020).

### Clinical Laboratory Variables

Seven standard clinical variables representing unique physiological functions or clinical feature were collected (serum albumin, ALB; total bilirubin, Bil; prothrombin time, PT; serum creatinine, Cr; ammonia, NH_4_; serum sodium, Na; and hepatic encephalopathy, HE) based on a previous study (Tan et al., 2020). HE was classified as unimpaired, minimal and overt HE according to Montagnese et al. (Montagnese et al., 2004; Vilstrup et al., 2014).

### Network Generation in the Population

**FIGURE 2:**
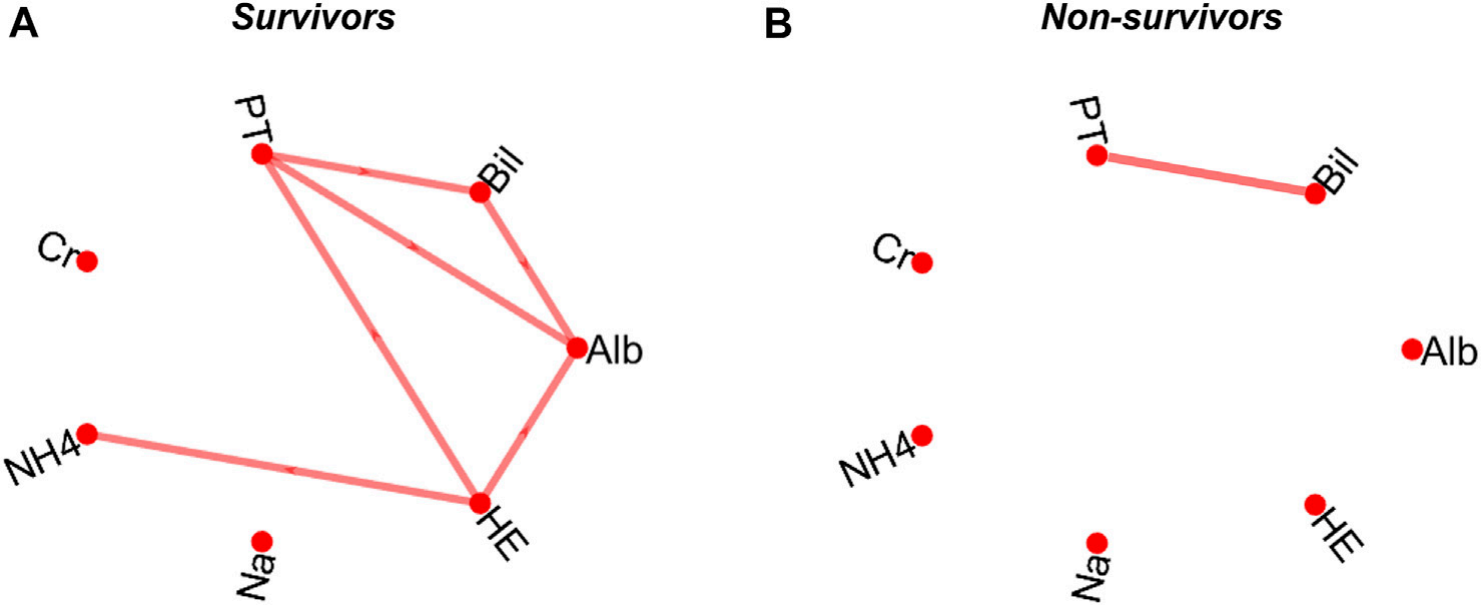
Correlation network map of survivors **(A)** and non-survivors **(B)** following a 12-month follow-up period. The map is based on a pairwise Spearman’s correlation’s correlation based on a Bonferroni-corrected significant level (*p* = 0.0024). serum albumin, Alb; total bilirubin, Bil; prothrombin time, PT; serum creatinine, Cr; ammonia, NH4; serum sodium, Na; and hepatic encephalopathy, HE.

The patients were grouped into two classes based on their survival status after 12 months of follow-up periods and a network map based on simple linear regression was constructed for both classes for visualisation (Tan et al., 2020). Nodes within network graphs represent clinical variables and the correlation between pairs of nodes are represented as edges. Spearman’s correlation was computed with pair matching to correct for missing data and the level of significance was based on a Bonferroni-corrected *p*-value (Dunn, 1961). An edge was formed between a pair of variables if the correlation between them is significant (*p* ≤ 0.0024: i.e., Bonferroni-corrected *p*-value). Pairs of clinical variables that did not meet the threshold for significant correlation in the correlation coefficient analysis were excluded from further analysis. The correlation network maps for survivors and non-survivors after a 12-month follow-up time are presented in Figures 2A,B.

### Parenclitic Network

#### Deviation Value Computation (δ)

The data of patients that survived after a 12-month follow-up period were used as reference in each follow-up time to construct a regression model based on the significantly correlated pairs of clinical variables above. As shown in Figure 2A, there is a significant correlation between 6 of the 21 computed pair of correlations in the survivor group. There is only one significant correlation between biomarkers in the non-survivor group (Figure 2B). The deviations between the data of all individual patients (survived and non-survived) from the pre-constructed reference model were then calculated as orthogonal residuals of the regression lines of each pair of correlated variables (Figures 1A–D).

#### Network Topology Analysis

Network topology analysis describes the underlying dynamics of a connected system. The network topology analysis of physiological functions have been shown to provide information on the adaptability and dynamic flexibility of organ systems to changes in the environmental conditions (Bashan et al., 2012). Several network topology metrics weighed by parenclitic deviations were used to assess the changes in physiological connectedness in patients with cirrhosis. These include network in-degree centrality, shortest path length, global diameter, and efficiency. Supplementary Table S1 presents the definitions and mathematical formulae of these indices.

#### Software Development

The software for computing the parenclitic network outputs was written in-house using MATLAB build R2021a (MATLAB, 2021) according to the originally described technique (Zanin et al., 2014). In summary, the software extracts and uses the data of the survivors to compute a Bonferroni-corrected regression model for all pairs of physiological variables (e.g., Na-Alb, Alb-Bil). The parameters (slope and intercepts) of significantly correlated pairs were used to compute the vertical and horizontal residuals (y and x respectively) which were then used to find the orthogonal residuals (delta, ∂) for all patients (survivors and non-survivors) as follows:

$$\;\partial =\frac{x.y}{\sqrt{x^{2}+y^{2}}}$$

The computed ∂’s were used as the weight of the connections between all correlated pairs of variables for all patients. Further, the individual ∂-weighted parenclitic network graphs are then used to perform the global network topology analyses. All computed results were combined into an output table which is labelled with the combined names of the variable pairs for the ∂’s and the computed network topology indices. The table is then written into a named, dated output saved in the workspace as a single excel file for further statistical analysis. The software is available in the GitHub repositor; https://github.com/topeoyelade.

### Statistical Analysis

Statistical analysis was performed using both MATLAB build R2021a (MATLAB, 2021) and SPSS Statistics 26 (IBM Corp., Armonk, New York) (Corp I.B.M., 2019). Initially, a Receiver Operating Curve (ROC) analysis was performed and the Area Under the Curve (AUC) was used to generate cut-off values that combines optimum sensitivity and selectivity in differentiation between the survivors and non-survivors for all computed output variables. Mann-Whitney U-test was used to compare the means of all output variables (∂’s and computed network topology indices) between the survivors and non-survivors. We performed Kaplan-Meier and log-rank (Mantel-Cox) test to assess whether the cut-offs from the ROC analysis can distinguish the groups. Further, bivariate Cox regression was computed to assess whether the significantly different variables with survival prediction can predict mortality independent of MELD and Child-Pugh scores. The combined prognostic index (e.g., MELD-∂) was calculated using the regression coefficients according to the following equation: $MELD-\partial \text{ index}=\beta_{1}\;MELD_{\;}\;+\;\beta_{2}\partial_{\;}\;$ where *β*_*1*_ and *β*_*2*_ are the regression coefficient of MELD and ∂ in bivariate Cox model respectively. Data are presented as median and interquartile range (IQR) and significant level was defined as two-tailed *p*-value < 0.05 in all analysis.

## Results

### Study Population

Overall, 106 patients diagnosed with cirrhosis were followed up for 12 months. During the follow up periods 17 deaths were recorded; 14 patients underwent transplantation due to liver failure or associated complications and were recorded as dead as they were considered to need a new liver to survive. The demography and clinical characteristics of the studied population is described in Table 1. Baseline biomarkers as well as MELD and Child-Pugh scores are presented in Supplementary Table S2 which shows a significant difference in most baseline biomarkers and MELD/Child-Pugh scores between survivors and non-survivors.

**TABLE 1:** Demographic and clinical variables in the study population.

	All patients (*n* = 106)
Age [Median (min–max)] (years)	58 (24–80)
Gender (male/female)	82/24
Aetiology of cirrhosis (alcohol/viral/others) (%)	42/34/24
MELD score [Median (min–max)]	12 (6–38)
Child-Pugh score [Median (min–max)]	8 (5–14)
Child class A/B/C	21/55/30

### Parenclitic Deviation (∂’s) of Survivors and Non-Survivors

Parenclitic deviation were compared between survivors and non-survivors and the results are shown in Tables 2. Based on Mann-Whitney U-test, there was increased parenclitic deviations in Alb-Bil (*p* < 0.001) and Alb-PT (*p* = 0.004) and Alb-HE (*p* = 0.034) axes compared with the non-survivors (Table 2).

**TABLE 2:** Comparison of parenclitic deviations of studied population.

∂ of variable pairs	Survivors; median (IQR)	Non-Survivors median (IQR)	*p*-value
Albumin-Bilirubin	2.08 (1.07–2.83)	5.09 (2.79–10.05)	**< 0.001**
Albumin-Prothrombin Time	2.48 (1.14–4.12)	4.83 (2.34–6.39)	**0.004**
Albumin-Hepatic Encephalopathy	0.50 (0.28–0.76)	0.63 (0.38–0.98)	**0.034**
Ammonia-Hepatic Encephalopathy	0.59 (0.25–0.80)	0.94 (0.30–1.28)	0.121
Bilirubin-Prothrombin Time	5.73 (3.58–8.90)	5.60 (2.31–11.49)	0.481
Hepatic Encephalopathy-Prothrombin Time	0.60 (0.16–0.88)	0.58 (0.10–0.98)	0.827

∂, parenclitic deviation; IQR, interquartile range.

### Parenclitic Deviations in Predicting Survival

Univariate Cox regression showed significant link between higher risk of mortality and parenclitic deviations along the Alb-Bil, Bil-PT, and the Ammonia-HE axes (Table 3). Higher deviation in the Alb-PT axis resulted in 20% increased risk of 12-month mortality (95% CI, 6%–35%, *p* < 0.001). Finally, deviation in the Alb-HE axis was linked with 3-fold increased risk of mortality after 12-month follow-up period (95% CI, 5% - 7-fold, *p* = 0.004: Table 3). A complete set of hazard ratios for all parenclitic deviations are shown in Supplementary Table S4.

**TABLE 3:** Univariate Cox regression analysis of the parenclitic deviations.

∂ of variable pairs	β	SEM	Hazard Ratio (95% CI)	*p*-value
Albumin-Bilirubin	0.128	0.024	1.137 (1.084–1.192)	**< 0.001**
Albumin-Prothrombin Time	0.179	0.062	1.195 (1.059–1.349)	**0.004**
Albumin-Hepatic Encephalopathy	1.005	0.487	2.732 (1.052–7.099)	**0.039**
Bilirubin- Prothrombin Time	0.030	0.006	1.030 (1.018–1.043)	**<0.001**
Hepatic Encephalopathy-Prothrombin Time	0.324	0.467	1.383 (0.554–3.451)	0.487
Ammonia-Hepatic Encephalopathy	1.369	0.606	3.933 (1.200–12.887)	**0.024**

∂, parenclitic deviation; β, coefficient of Cox regression analysis; SEM, standard error of mean of β, CI, confidence interval.

### Independence of Parenclitic Deviations in Predicting Survival

To assess whether the ability of the parenclitic deviations to significantly predict survival is independent of the index of liver disease severity (MELD), we performed bivariate Cox regressions for parenclitic deviations with MELD as covariate. The parenclitic deviation along the Alb-Bil (Hazard Ratio, 95% CI = 1.063, 1.000–1.129; *p* = 0.048) and Alb-PT (Hazard Ratio, 95% CI = 1.138, 1.012–1.280; *p* = 0.031) axes predicted 12-month survival independent of MELD (Table 4). To study this further, we looked at the independence of parenclitic deviations from Child-Pugh score, a classic measure for severity of hepatic dysfunction. Our results showed that parenclitic deviation of the Alb-Bil, Alb-PT and Bil-PT predicted 12-month survival independent of Child-Pugh scores (Supplementary Table S3).

**TABLE 4:** **–** The prognosis effects of parenclitic deviations independent of MELD using bivariate Cox regression analysis.

∂ with MELD	β	SEM	Hazard Ratio (95.0% CI)	*p*-value
Albumin-Bilirubin	0.061	0.031	1.063 (1.000–1.129)	**0.048**
MELD	0.119	0.038	1.126 (1.047–1.213)	**0.002**
Albumin-Prothrombin Time	0.129	0.060	1.138 (1.012–1.280)	**0.031**
MELD	0.435	0.092	1.166 (1.109–1.251)	**<0.001**
Albumin-Hepatic Encephalopathy	0.702	0.501	2.017 (0.756–5.383)	0.161
MELD	0.152	0.030	1.164 (1.099–1.229)	**<0.001**
Bilirubin-Prothrombin Time	0.013	0.008	1.014 (0.997–1.030)	0.101
MELD	0.143	0.033	1.153 (1.082–1.229)	**<0.001**
Ammonia-Hepatic Encephalopathy	1.093	0.628	2.983 (0.870–10.219)	0.082
MELD	0.138	0.032	1.148 (1.078–1.223)	**<0.001**

∂, parenclitic deviation; β, coefficient of Cox regression analysis; SEM, standard error of mean of β, Ci, confidence interval; MELD, Model for End-stage Liver Disease.

### Receiver Operating Characteristics Curves of Parenclitic Deviations

**FIGURE 3:**
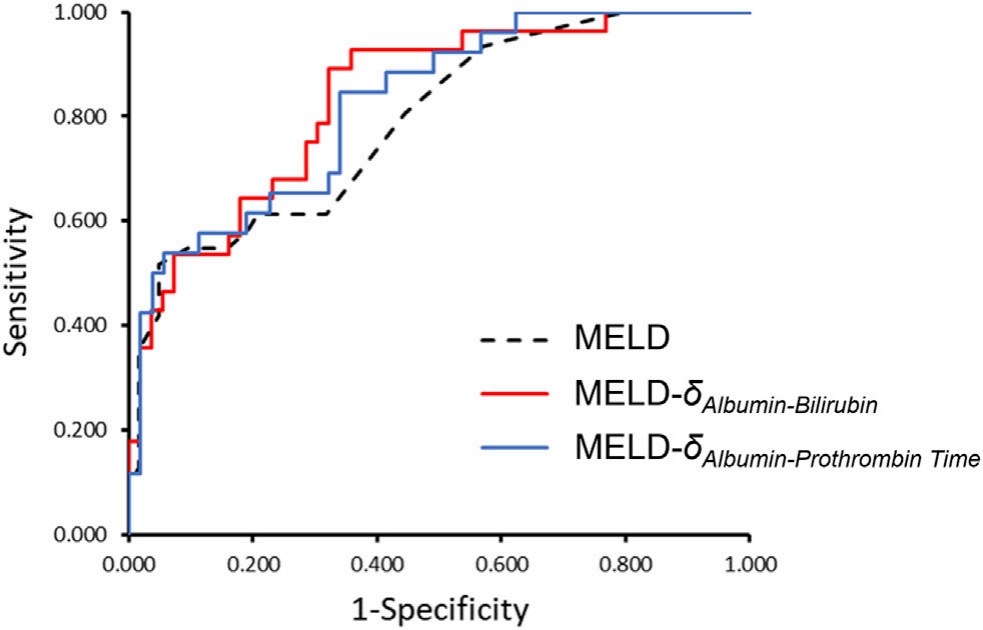
The ROC curves comparing MELD alone with MELD-δ_Alb-Bil_ and MELD- δ_Alb-PT_ in classifying patients as survivor or non-survivor. Addition of parenclitic deviation of Alb-Bil and Alb-PT axes could increase the AUC for MELD from 0.792 (95% CI, 0.696–0.888) to 0.835 (0.747–0.924) and 0.824 (0.730–0.918) respectively (*p* < 0.001 for all curves).

ROC curves were computed for the parenclitic deviations for 12-month follow-up periods that predicted survival independent of MELD (Table 5). The deviation along the Alb-Bil axis showed similar AUC in comparison with MELD (0.762 versus 0.792). As shown in Figure 3 and Table 5, addition of parenclitic deviation of Alb-Bil and Alb-PT axes could increase the AUC for MELD from 0.792 to 0.835 and 0.824 respectively (*p* < 0.001).

**TABLE 5:** Area under the ROC curves (AUC) of parenclitic deviations (∂), MELD and combined MELD-∂ during 12-month follow-up periods.

Prognostic index	AUC (95% CI)	*p*-value
Albumin-Bilirubin	0.762 (0.652–0.872)	**< 0.001**
Albumin-Prothrombin Time	0.696 (0.569–0.824)	**0.004**
MELD	0.792 (0.696–0.888)	**< 0.001**
MELD-**∂**_Albumin-Bilirubin_	0.835 (0.747–0.924)	**< 0.001**
MELD-**∂**_Albumin-Prothrombin Time_	0.824 (0.730–0.918)	**< 0.001**

CI, confidence interval; AUC, area on the receiver operating curve.

### Kaplan-Meier Graphs of Parenclitic Deviations

**FIGURE 4:**
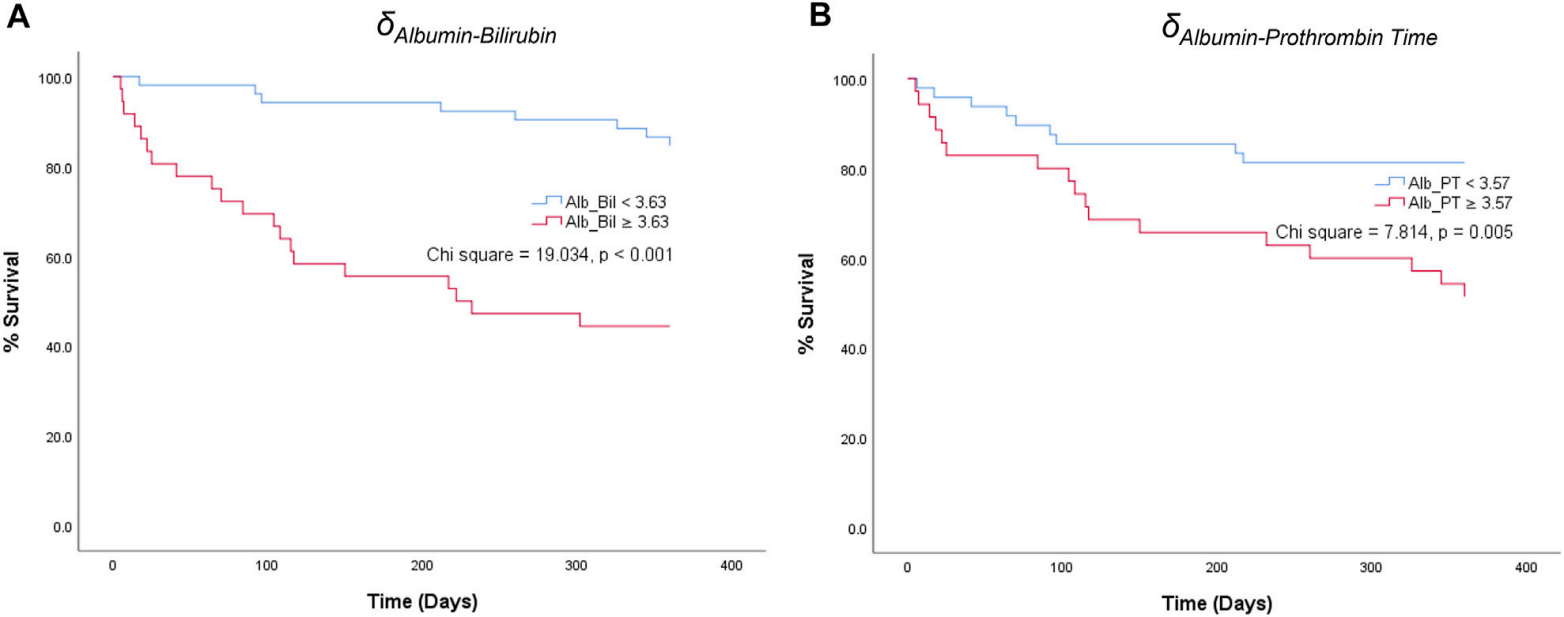
Kaplan-Meier graphs showing 12-month survival predictions of parenclitic deviations along the **(A)** Albumin-Bilirubin (Alb_Bil) and **(B)** Albumin-Prothrombin Time (Alb_PT) axes based on the cut-off values of 3.63 and 3.57 respectively [Log-rank (Mantel-Cox) test, Chi square = 19.034, *p* < 0.001 and 7.814, *p* = 0.005 respectively].

**FIGURE 5:**
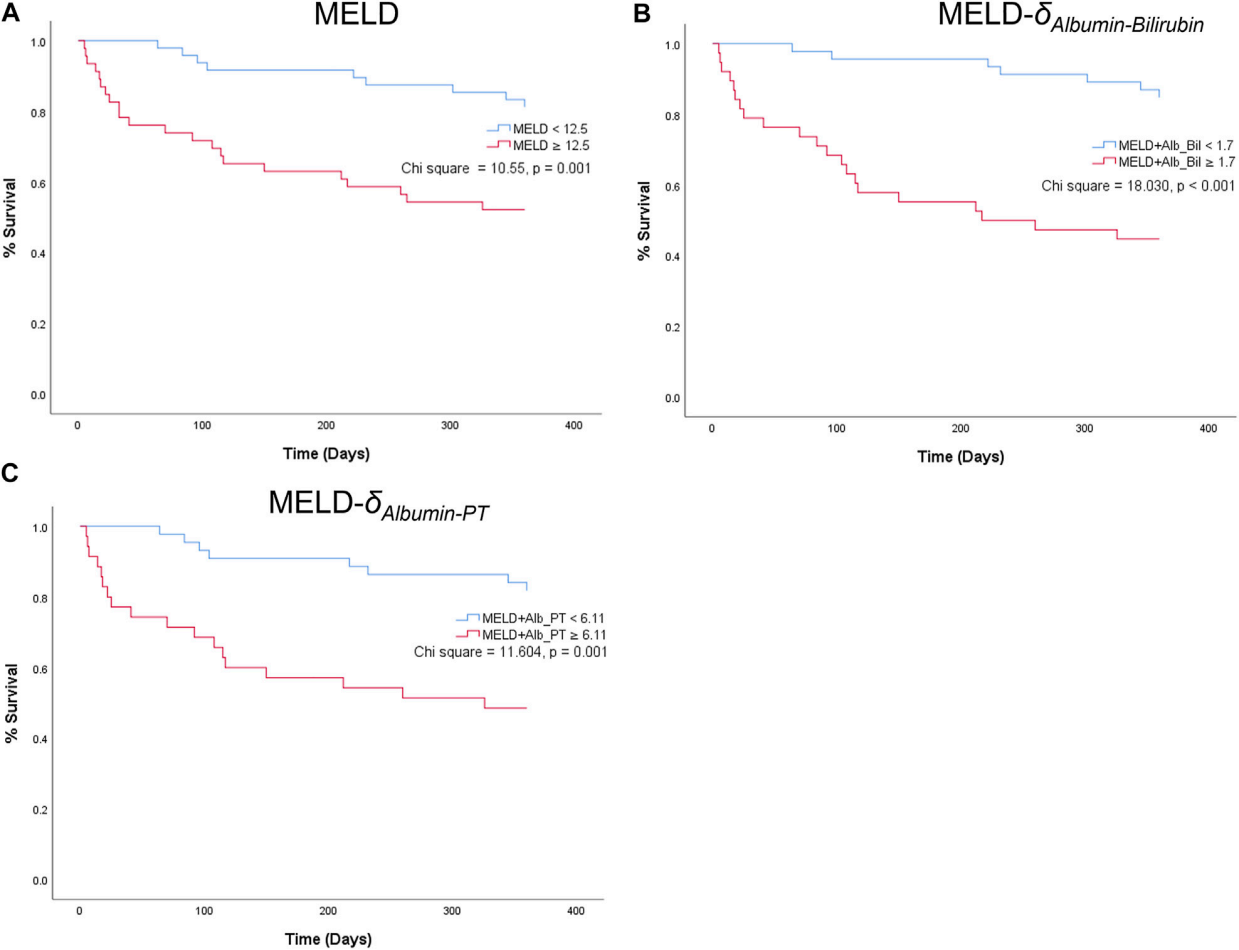
Kaplan-Meier graphs showing 12-month survival predictions of MELD **(A)** and two combined indices: MELD-δ_Albumin-Bilirubin_ (MELD-Alb_Bil) **(B)** and MELD--δ_Albumin-PT_ (MELD-Alb_PT) **(C)**.

For the parenclitic deviations that were significantly predictive of survival independent of MELD, cut-offs with the optimum sensitivity and specificity were generated from their ROC curves (i.e., optimum sensitivity and specificity for prediction of survival). The deduced cut-offs were then used to group the patients into group “predicted non-survivor” if the patients’ parenclitic deviations are higher than or equal to the corresponding cut-off values or “predicted survivor” if otherwise. The binary output was then used to generate Kaplan-Meier graphs to assess the prognostic value. Figure 4 indicates that both Alb-Bil and Alb-PT deviations can predict 12 months survival with a statistically significant log-ranked test (Chi-square 19.03 and 7.81 respectively). Furthermore, addition of Alb-Bil or Alb-PT deviations to MELD in a bivariate Cox regression model enhances the prognostic value of MELD alone (Figure 5).

### Network Topology Indices and Prediction of Survival in Patients With Cirrhosis

A shown in Supplementary Table S4, there was significant increase in standard deviation of centrality between the survivors and non-survivor group (*p* = 0.038). Other topology indices did not exhibit statistically significant difference. Cox regression analysis was performed to determine the relationship between network topology indices and survival. Higher standard deviation of centrality increased the risk of mortality with a hazard ratio of 1.054 (95% CI, 1.026–1.083, *p* < 0.001). Furthermore, the standard deviation of centrality was able to predict survival independent of Child-Pugh score (Supplementary Table S5).

## Discussion

In this study, a parenclitic approach was used to map the physiological network of patients with cirrhosis from routine clinical/laboratory data. By using the data of survivors to construct a reference model, deviations for each patient’s pairs of variables from the reference model were calculated and used for prognosis calculation. We found that increased parenclitic deviations and reduced connectedness in the Ammonia-HE axis is associated with ∼4-fold increase in the risk of mortality. Reduced connectedness along the Alb-Bil, Alb-PT and Bil-PT axes were also linked with increased risk of mortality independent of routine prognostic indices such as MELD and Child-Pugh. Higher parenclitic deviations shown by non-survivors suggests a digression from the expected connection along various physiological axes and can be interpreted as significant network disruption between organ systems (i.e., more parenclitic deviation = less organ systems connectivity).

Furthermore, we analysed the network topology indices characterising the parenclitic networks defined by weighted deviations. This gives quantitative measure of the network and evaluates the deviations and their collective relationships. From a set of topological indices, standard deviation of centrality was significantly higher in the non-survivors than survivors and showed significant association with 12-month survival. This global index was also observed to predict survival independent of Child-Pugh. Put together, these results show that parenclitic network analysis can detect certain functional dynamics not picked up by the current models used for prognostication in cirrhosis. These results highlight the significance of interrelationships between clinical variables such as Alb-Bil, Alb-PT and Ammonia-HE in reflecting the pathological stage of cirrhosis and provides insight into complex interactions between extrahepatic complications manifested in multiple organ systems and how they may exacerbate the prognosis of patients with cirrhosis.

A network approach to complex disease such as liver failure has potential to transform the landscape of assessing prognosis. The present study indicates that a parenclitic approach with routine laboratory tests (e.g., albumin, bilirubin, PT) may increase the accuracy of current prognostic factors and be used in conjunction with MELD to ultimately increase the number of lives saved. This is in line with previous research revealing other physio-markers such as EEG or heart rate variability (HRV) in conjunction with MELD to increase the accuracy of prognostication (Montagnese et al., 2015; Chan et al., 2017; Bhogal et al., 2019; Oyelade, 2021). However, while analysis of EEG or HRV requires suitable recording equipment and analytical expertise, the parenclitic approach introduced in this study uses routine laboratory tests that is available in all clinical settings. This is an advantage for this approach and can be extended in future multi-centre prospective clinical investigations. Such a network approach also has potential to be used in other complex illnesses such as sepsis and multiple organ failure for survival modelling as well as providing novel insight about the pathophysiology. If organ systems network disruption plays an important role in critically ill patients (Asada et al., 2016), novel therapies may target enhanced levels of connectivity of organ systems rather than treating functional systems in isolation using pharmacological antagonists.

Our results indicate that parenclitic deviation from albumin-bilirubin, albumin-PT and ammonia-HE axes provide useful information for prognostication. Hepatic encephalopathy is a spectrum of neurophysiological disturbances that occurs in the background of acute or chronic liver failure (Aldridge et al., 2015). Although classically linked with hyperammonaemia, systemic inflammation is known to precipitate or cause exacerbation of HE (Shawcross et al., 2011; Tranah et al., 2013). While the exact link between systemic inflammation, ammonia and HE remain unclear, systemic inflammation (due to endotoxemia, or bacterial translocation) may increase the susceptibility of the brain to hyperammonaemia thereby derailing the correlation between increased serum ammonia and HE. While there was a positive correlation between ammonia and HE in survivors (r = 0.469, *p* = 0.002), the severity of HE was not significantly associated with ammonia in non-survivors (r = −0.027, *p* = 0.911). This show that factor(s) other than ammonia may be contributing to HE in non-survivors. Indeed, various studies have linked systemic inflammation with increased severity and poorer prognosis of HE (Rolando, 2000; Vaquero et al., 2003; Shawcross et al., 2004; Shawcross et al., 2007; Sharifi, 2008). Thus, the increased parenclitic deviation along the Ammonia-HE axis may reflect the contribution of a secondary physiological factor which predisposes an increased mortality from cirrhosis. This can be easily analysed using a parenclitic approach as described here or more traditional statistical methods such as analysis of covariance.

Our analysis showed that the correlation between albumin and bilirubin is lost in non-survivors. There was a sharp reduction in serum albumin with increased bilirubin in survivors compared to non-survivors (Supplementary Figure S1). The reason for this disruption is not well clear. However, we hypothesis that; 1) The relatively high albumin observed even at significantly elevated bilirubin level in non-survival may be due to clinical infusion which may not improve the effective systemic albumin or prognosis (Solà et al., 2018; Fernández et al., 2020) but may be associated with increased serious adverse events as was recently reported in the ATTIRE study (China et al., 2021); 2) The half-life of albumin is comparatively higher at about 3 weeks (Peters, 1995) compared to bilirubin which remains in circulation for about 6 min (Reed et al., 1988). In addition, the half-life of albumin might be altered in critically ill patients due impaired microcirculation compared with healthier patients (Vincent et al., 2014) a factor that may contribute to difference in albumin-bilirubin correlation or survivors and non-survivors.

Albumin-PT was another axis that differentiated survivors from non-survivors in our study. The liver produces most procoagulant and anticoagulant proteins, responsible for maintaining haemostasis. In cirrhotic patients, the production of clotting factors and their inhibitors decreases, results in either a “rebalanced” haemostatic equilibrium or a prothrombotic state due to systemic inflammation (Baccouche et al., 2017). Increased bleeding risk has traditionally been regarded as the most significant haemostatic complication in patients with liver dysfunction, especially in the context of elevated international normalized ratio (INR) (Flores et al., 2017). However, the predictive value of INR in indicating the risk of haemorrhagic event has been contradicted in literature and remains unclear (Lisman and Leebeek, 2007; Lisman and Porte, 2010). On the contrary, there is an increasing recognition of hypercoagulability in some patients with cirrhosis where the risk of thrombotic events (e.g., portal vein thrombosis) might be higher than haemorrhage (Tripodi et al., 2011; Tripodi et al., 2017; Talon et al., 2020). Portal vein thromboses and clotting of extracorporeal circuits are common in cirrhosis despite elevated INR values, while elevated bleeding tendency has been suggested to be associated with sepsis, hepatorenal syndrome, hypotension, and endothelial dysfunction instead of isolated liver dysfunction (Harrison, 2018). Indeed, venous thromboembolism (VTE) is an underdiagnosed and serious medical condition that occurs at a relative risk of >2% in cirrhotic patients and associated with greater mortality in higher Child-Pugh stages (Buresi et al., 2012; Yang et al., 2012). Also, low serum albumin has been found to be strongly predictive of increased risk of VTE, independent of INR or platelet account (Northup et al., 2006). It is hypothesized that lower serum albumin concentration is a surrogate for decreased protein synthesis by the liver and therefore correlated with decreased production of endogenous anti-coagulant factors such as Protein C and S. Our results share similar findings (Supplementary Figure S2), that the albumin levels are generally lower in non-survivors and remain low despite increase in PT. While in survivors, albumin levels present positive linear increase with PT. In cirrhosis, coagulopathy involves a complicated network of haemostatic factors, with the risks of thrombotic and haemorrhagic events reported to be independent of current markers or scores (Harrison, 2018). Therefore, a parenclitic approach to relationship between albumin and PT might pave the way for assessment of this relationship in routine clinical practice.

This study validates the feasibility of parenclitic network-based approach for predicting the survival status of patients with liver cirrhosis, and its independence from Child-Pugh and MELD scores, indicating that including the correlation between biomarkers improves current prognostic indices and may help improve the accuracy of prognostication. This suggests that a parenclitic approach has potential in complementing current prognostic scoring systems for liver cirrhosis. However, there are some limitations. Firstly, the data of survivors was used as reference for measurement of deviations is limited in size and from a single referral medical centre. Future studies need to look at a more diverse multicentre cohort of patients with cirrhosis. Full applicability of the parenclitic method in survival modelling in cirrhosis requires further validation by applying the reference parameters developed in the current study to an external dataset of patients with cirrhosis. Alternatively, constructing parenclitic networks in bootstrap replica of the data may provide further information on reliability of this approach. Further studies can investigate validation of such a network approach in a larger and more clinically diverse patient population. Another limitation of this study is that the relationship models of different clinical variables were based on linear regression, which assumes a correlative linear relationship between all pairs of variables. More sophisticated methods such as the 2-dimensional kernel density estimation (Whitwell et al., 2018) could potentially serve as a better approach, as it provides compatibility of categorical and continuous data. Further, various variables such as inflammatory biomarkers (e.g., IL-6) and physiological markers (e.g., heart rate variability, heart rate turbulence and temperature variability indices), that were shown to predict mortality in cirrhosis patients (Mani et al., 2009; Bottaro et al., 2020) could be included in the analysis to widen the scope and improve the prognostic value of the parenclitic method. In addition, the results of this study might not be extendable to all subgroup of patients with cirrhosis as data were selected from patients referred to a tertiary referral clinic for evaluation of HE. For example, the parenclitic network may exhibit a different pattern in patients with acute-on-chronic liver failure (ACLF) compared with other forms of decompensation. This may give insight about the mechanism of decompensation and organ failure in cirrhosis. Future studies can focus on more diverse, and clinically relevant subgroups of patients with cirrhosis to provide a more comprehensive picture of organ systems network disruption in individual patients with cirrhosis. The present study also lacks a time-dependent approach in predicting outcome using the parenclitic networks. Assessment of network structure over time can provide useful information on the trajectory of alterations in physiological processes involved in decompensation and might be of significant value for prognosis evaluation.

In conclusion, this is the first study to use the parenclitic network analysis of routine clinical data to assess organ system disruption and predict survival in individual patients with cirrhosis. Potential application of this method includes the prediction of treatments response or patients likely to develop serious adverse events due to certain treatments. For example, patients with decompensated cirrhosis indicated for vasoconstrictors and/or albumin treatment who may not respond (Cavallin et al., 2015; Wang et al., 2018; Moore et al., 2020) or those likely to develop respiratory failure (Wong et al., 2021) or other side effects (Martín–Llahí, 2008; Neri et al., 2008; Sanyal et al., 2008; Gluud et al., 2010). The holistic approach of the parenclitic network analysis may prove to be a better prognostic method and can provide novel pathophysiologic insight for understanding complex diseases such as chronic liver failure.

## Data Availability

The raw data supporting the conclusion of this article will be made available by the authors, without undue reservation.
