# Supplementary material for: Prognosis and Survival Modelling in Cirrhosis Using Parenclitic Networks
Source: Front Netw Physiol. 2022 Feb 21;2:833119. doi: 10.3389/fnetp.2022.833119 (PMC10013061; doi:10.3389/fnetp.2022.833119)
Supplement: Supplementary file 1 [file Table1.docx]

**Supplemental Materials**

**Table S1 – Description of network topology measures**

| **Network Topology Measures** | **Definition** | **Mathematical Formula** |
| --- | --- | --- |
| Degree Centrality | Sum of incidental edges to the node. In directed network, it is represented as the sum of weighted incoming or outcoming edges of each node. | $D\left( i \right)=\sum_{j=1}^{n} A_{ij}$  -$A_{ij}$ is the *ij*-th element of the adjacency matrix A of the graph  -n is the number of nodes in the graph [1]. |
| Shortest Path Length (SPL) | A path with the minimum number of edges between two nodes. In directed and weighted network, it is a path with the minimum sum of edge weights starting at source node s and ending at target node t. | $d(i,j)$  -distance *d(i,j)* denotes the length of the shortest path between node i and node j |
| Efficiency | The efficiency between two nodes is defined as reciprocal of their shortest path length. The global efficiency of a graph is the average efficiency over all pair of nodes. | $E\left( G \right)=\frac{1}{n(n-1)}\sum_{ij} \frac{1}{d(i,j)}$  [2] |
| Diameter | The shortest path length between the most distant nodes. | $d(G)=\begin{matrix} max \\ ij \end{matrix}\{d\left( i,j \right)\}$ |

**References**

1. Srinivasan, S., et al., *Chapter Three - Machine learning techniques for fractured media*, in *Advances in Geophysics*, B. Moseley and L. Krischer, Editors. 2020, Elsevier. p. 109-150.

2. Ek, B., C. VerSchneider, and D.A. Narayan, *Global efficiency of graphs.* AKCE International Journal of Graphs and Combinatorics, 2015. **12**(1): p. 1-13.

**Table S2. Comparison of circulating biomarkers, MELD and Child-Pugh scores in survival and non-survival patients with cirrhosis after one year follow up.**

| **Variable** | **Survivors** | **Non-Survivors** | **p-value** |
| --- | --- | --- | --- |
| Albumin (g/L) | 34.9 (30.8 – 38.1) | 30.0 (27.1 – 36.1) | **0.005** |
| Bilirubin (µmol/L) | 23.1 (15.4 – 38.2) | 73.9 (31.1 – 141.8) | **<0.001** |
| Prothrombin time (% activity) | 54 (48 - 67) | 46 (32 – 55) | **<0.001** |
| Ammonia (µmol/L) | 59.5 (35.7 – 115.0) | 60 (29.0 – 85.3) | 0.266 |
| Creatinine (µmol/L) | 74 (65 - 93) | 89 (70 – 115) | **0.039** |
| Sodium (mmol/L) | 138 (136 – 140) | 136 (134 – 138) | **0.008** |
| MELD score | 11 (9 – 14) | 19 (12 – 23) | **<0.001** |
| Child-Pugh score | 7 (6 – 9) | 10 (8 – 11) | **<0.001** |

*Data are shown as Median (interquartile range). P-values are calculated using Mann-Whitney U test.*

**Table S3. The prognosis effects of parenclitic deviations independent of Child-Pugh score.**

| **∂ of variable pairs** | **β** | **SEM** | **Hazard Ratio (95.0% CI)** | **p-value** |
| --- | --- | --- | --- | --- |
| Albumin-Bilirubin  Child-Pugh | 0.095  0.430 | 0.025  0.098 | 1.100 (1.048 – 1.154)  1.537 (1.269 – 1.861) | **<0.001**  **<0.001** |
| Albumin-Prothrombin Time  Child-Pugh | 0.106  0.153 | 0.063  0.032 | 1.162 (1.016 – 1.327)  1.545 (1.291 – 1.849) | **0.028**  **<0.001** |
| Albumin-Hepatic Encephalopathy  Child-Pugh | 0.569  0.431 | 0.515  0.090 | 1.766 (0.644 – 4.844)  1.539 (1.289 – 1.837) | 0.269  **<0.001** |
| Bilirubin-Prothrombin Time  Child-Pugh | 0.024  0.466 | 0.007  0.095 | 1.024 (1.010 – 1.038)  1.594 (1.324 – 1.091) | **0.001**  **<0.001** |
| Ammonia-Hepatic Encephalopathy  Child-Pugh | 0.651  0.456 | 0.673  0.112 | 1.918 (0.513 – 7.171)  1.578 (1.268 – 1.963) | 0.333  <0.001 |

*∂, parenclitic deviation; β, coefficient of Cox regression analysis; SEM, standard error of mean of β, CI, confidence interval.*

**Table S4. Parenclitic network topology of studied population.**

| **Network topology** | **Survivors; median (IQR)** | **Non-Survivors; median (IQR)** | **p-value** |
| --- | --- | --- | --- |
| Centrality Sum | 25.61 (15.98 – 31.58) | 32.20 (21.82 – 51.05) | 0.070 |
| Centrality Mean | 3.66 (2.28 – 4.51) | 4.60 (3.12 – 7.29) | 0.070 |
| Centrality Standard Deviation | 5.88 (3.64 – 8.61) | 8.71 (4.57 – 13.88) | **0.038** |
| Shortest Path Length Sum | 17.88 (12.28 – 24.45) | 21.86 (15.70 – 34.11) | 0.202 |
| Shortest Path Length Mean | 2.55 (1.75 – 3.49) | 3.12 (2.24 – 4.87) | 0.202 |
| Shortest Path Length Standard Deviation | 2.40 (1.61 – 3.91) | 2.99 (1.91 – 4.40) | 0.446 |
| Efficiency | 0.03 (0.02 – 0.05) | 0.03 (0.02 – 0.04) | 0.303 |
| Diameter | 6.11 (4.23 – 9.79) | 6.77 (5.05 – 11.35) | 0.303 |

IQR, Interquartile range.

**Table S5 – The prognosis effects of parenclitic deviations independent of MELD and Child-Pugh score.**

| **Network topology** | **β** | **SEM** | **Hazard Ratio (95.0% CI)** | **p-value** |
| --- | --- | --- | --- | --- |
| Standard Deviation of Centrality  MELD | 0.021  0.112 | 0.018  0.043 | 1.022 (0 .986 – 1.058)  1.119 (1.029 – 1.216) | 0.231  **0.009** |
| Standard Deviation of Centrality  Child-Pugh | 0.037  0.539 | 0.014  0.122 | 1.037 (1.010 – 1.066)  1.714 (1.350 – 2.176) | **0.008**  **<0.001** |

*β, coefficient of Cox regression analysis; SEM, standard error of mean of β, CI, confidence interval.*

**Fig. S1 –** Graph showing relationship between albumin and bilirubin in patients that were followed up for 12 months. The red and green dots represent the data of the non-survivors and survivors respectively with the corresponding lines depicting the direction of correlation. Analysis of covariance (ANCOA) was applied to assess the effect of group (survivors versus non-survivor) in the relationship between albumin and bilirubin. ANCOVA showed F(group)= 4.215, P<0.05. This provides statistical evidence that there are statistically significant differences in albumin levels between the groups (i.e., survivors and non-survivors) when adjusted for patients’ bilirubin level.


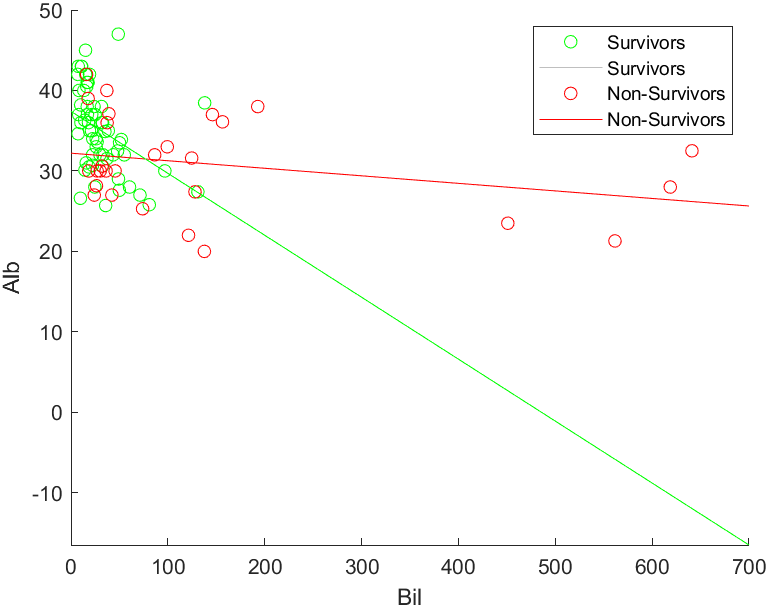


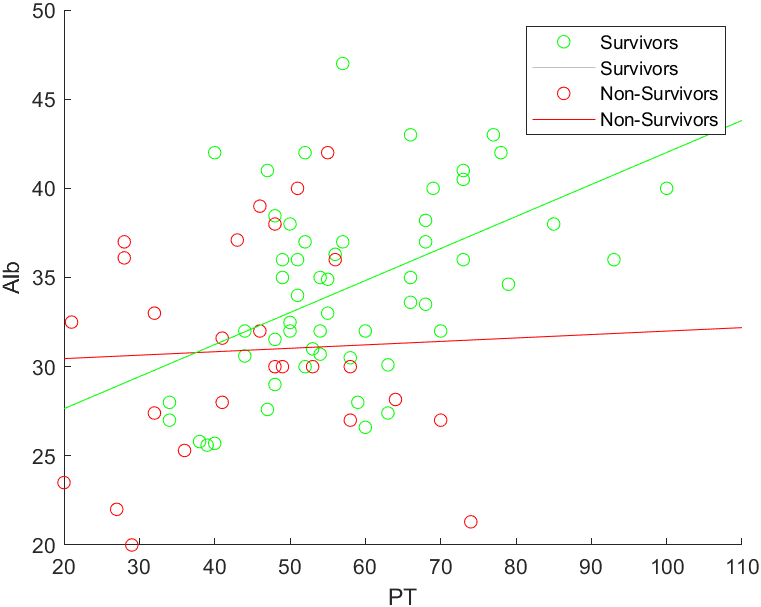
**Figure S2 –** Graph showing relationship between albumin and prothrombin time in patients that were followed up for 12 months. The red and green dots represent the data of the non-survivors and survivors respectively with the corresponding lines depicting the direction of correlation.
